# Effectiveness of Patient Navigation During Transition to Adult Care: A Randomized Clinical Trial

**DOI:** 10.1001/jamapediatrics.2024.6192

**Published:** 2025-02-10

**Authors:** Susan Samuel, Zoya Punjwani, Daniella San Martin-Feeney, Brooke Allemang, Gregory M.T. Guilcher, Eddy Lang, Danièle Pacaud, Jorge Pinzon, Gail Andrew, Lonnie Zwaigenbaum, Curtis Perrott, John Andersen, Lorraine Hamiwka, Alberto Nettel-Aguirre, Scott Klarenbach, Kerry McBrien, Shannon D. Scott, Megan Patton, Sophie Samborn, Ken Pfister, Laurel Ryan, Gina Dimitropoulos, Andrew S. Mackie

**Affiliations:** 1 Department of Pediatrics, Cumming School of Medicine, University of Calgary, Calgary, Alberta, Canada; 2 Department of Community Health Sciences, Cumming School of Medicine, University of Calgary, Calgary, Alberta, Canada; 3 Alberta Children’s Hospital Research Institute, Calgary, Alberta, Canada; 4 Department of Pediatrics, University of Alberta, Edmonton, Alberta, Canada; 5 Faculty of Social Work, University of Calgary, Calgary, Alberta, Canada; 6 Child Health Evaluative Sciences, SickKids Research Institute, Toronto, Ontario, Canada; 7 Section of Pediatric Oncology and Blood and Marrow Transplant, Cumming School of Medicine, University of Calgary, Calgary, Alberta, Canada; 8 Department of Emergency Medicine, Cumming School of Medicine, University of Calgary, Calgary, Canada; 9 Glenrose Rehabilitation Hospital, Edmonton, Alberta, Canada; 10 School of Mathematics and Applied Statistics, Faculty of Engineering and Information Services, University of Wollongong, Wollongong, New South Wales, Australia; 11 Department of Medicine, University of Alberta, Edmonton, Canada; 12 Department of Family Medicine, Cumming School of Medicine, University of Calgary, Calgary, Alberta, Canada; 13 Faculty of Nursing, University of Alberta, Edmonton, Alberta, Canada; 14 Mathison Centre for Mental Health Research and Education, Calgary, Alberta, Canada; 15 Stollery Children’s Hospital, Edmonton, Alberta, Canada

## Abstract

**Importance:**

Transition to adult care is a challenging and complex process for youth and emerging adults with chronic health and/or mental health conditions. Patient navigation has been proposed to improve care during transition, but previous studies have used single disease cohorts with a nonrandomized design.

**Objective:**

To compare the effectiveness of a patient navigator service to reduce emergency department (ED) use among adolescents and emerging adults with chronic health and/or mental health conditions undergoing transition to adult-oriented health care.

**Design, Setting, and Participants:**

This was a pragmatic, parallel-group, nonblinded randomized clinical trial design. Patients were followed up for a minimum 12 months and maximum 24 months after enrollment. The setting was the Canadian province of Alberta, with a population of 4.3 million inhabitants, having 3 tertiary care pediatric hospitals serving the entire population with universal health coverage. Participants included youth aged 16 to 21 years, followed up within a diverse array of chronic care clinics, expected to be transferred to adult care within 12 months, residing in Alberta, Canada.

**Interventions:**

A 1:1 allocation to either access to a personalized navigator, an experienced social worker within the health services environment, or usual care, for up to 24 months after randomization.

**Main Outcomes and Measures:**

All-cause ED visit rate while under observation.

**Results:**

A total of 335 participants were randomized over a period of 45 months, 164 (49.0%) to the intervention arm and 171 (51.0%) to usual care. After 1 patient withdrew, 334 participants (usual care: mean [SD] age, 17.8 [0.7] years; 99 female [57.9%]; intervention: mean [SD] age, 17.7 [0.6] years; 81 male [49.7%]) were included in the final data analysis. Among the participants, 131 (39.2%) resided in a rural location, and 126 (37.7%) had a self-reported mental health comorbidity during baseline assessment. We observed significant effect modification in the relationship between intervention and ED visits based on mental health comorbidity. Among those with a self-reported mental health condition, ED visit rates were lower in those with access to the navigator, but the association was not significant (adjusted incidence rate ratio [IRR] 0.75; 95% CI, 0.47-1.19). Among those with no mental health comorbidity, the corresponding adjusted IRR was 1.45 (95% CI, 0.95-2.20).

**Conclusions and Relevance:**

In this randomized clinical trial, the navigator intervention was not associated with a significant reduction in ED visits among youth with chronic health conditions transitioning to adult care. The study did not accrue sufficient sample size to demonstrate a significant difference between groups should it exist.

**Trial Registration:**

ClinicalTrials.gov Identifier: NCT03342495

## Key Points

**Question:**

Is the rate of emergency department visits for adolescents and emerging adults with chronic health conditions during transition to adult-oriented health care lower for patients with access to a patient navigator than for those receiving usual care?

**Findings:**

This parallel-group randomized clinical trial enrolled 334 participants followed up in chronic care clinics in Alberta, Canada. Among participants with a mental health comorbidity, those who received support from the patient navigator had fewer emergency department visits compared with those receiving usual care; this relationship was not statistically significant.

**Meaning:**

Results suggest that the navigator intervention may not reduce emergency department visits in this population.

## Teaser

This randomized clinical trial investigates if the rate of emergency department visits for adolescents with chronic health conditions during the transition to adult health care is lower for patients with access to a patient navigator than for those receiving usual care.

## Introduction

Adolescents and emerging adults (AEAs) living with chronic physical or mental health conditions undergo the transfer from pediatric to adult health care.^1^ This occurs at a single point in time wherein there is often a lack of coordination and preparation. Transfer of care occurs at a vulnerable developmental period during which there is often poor treatment adherence, increased emergency department (ED) visits, and poor overall health outcomes.^1,2,3,4,5,6^ AEAs have expressed that the transfer to adult care is a difficult time due to lack of resources in some jurisdictions and lack of personnel to help navigate AEAs to adequate resources and supports. The heightened expectations of self-management of health conditions in adult health care settings differs vastly from the health care and parental/guardian supports present in pediatric care environments. The need for additional supports for AEAs at the time of transfer and transition period is undisputed.^1,6,7,8^

Patient navigators (PNs) are a promising intervention to facilitate planned transitions from pediatric to adult care and improve patient experience and outcomes. A limited number of observational studies have shown that access to a navigator during transition decreases dropout from medical care and disease-specific adverse events.^9,10^ The evidence base for PN interventions during transition to adult care is limited by a paucity of data from controlled studies.

We conducted a pragmatic parallel-group randomized clinical trial (RCT), the Transition Navigator Trial (TNT).^11^ The primary objective was to evaluate the impact of a personalized transition to adult care intervention (ie, access to a navigator) on ED/urgent care visit rates for individuals aged 16 to 21 years living with chronic health or mental health conditions.

## Methods

### Trial Design, Setting, and Participants

The TNT study used a parallel-group, pragmatic RCT design. Eligible participants were recruited from 41 pediatric specialty clinics within 3 tertiary care pediatric hospitals: Alberta Children’s Hospital, Stollery Children’s Hospital, and Glenrose Rehabilitation Hospital. These hospitals are responsible for all tertiary specialty pediatric health care in the province of Alberta, Canada. Transfer to adult care generally occurs around age 18 years in Alberta, and transitional care varies by clinic and efforts by individual staff members.

Ethics approval was obtained from the University of Calgary Conjoint Health Research Ethics Board and the University of Alberta Health Research Ethics Board. The study protocol was published in 2019 (Supplement 1).^11^ This report was created according to standards set out in the (1) Consolidated Standards of Reporting Trials (CONSORT) 2010 reporting guidelines, ^12^ (2) CONSORT and SPIRIT Extension for RCTs Revised in Extenuating Circumstances (CONSERVE) 2021 statement,^13^ and (3) the better reporting of interventions template for intervention description and replication (TIDieR) checklist.^14^

#### Inclusion Criteria

We included patients aged 16 to 21 years having 1 or more chronic health conditions (including mental health) lasting longer than 3 months, requiring referral to an adult-oriented specialist,^7,8^ and having a last planned pediatric visit within 12 months of enrollment.

#### Exclusion Criteria

Patients were excluded if they were enrolled in another transition-related study involving a PN or similar intervention, if there was no planned transfer to another center or physician, or if they planned to move out of Alberta during the study period.

#### Ethics and Consent

Written or oral informed consent was obtained from all participants. Consent was also sought from 1 parent/guardian. If the participant refused parent/guardian involvement in the study, they were not contacted during the intervention nor was health information provided to the parent/guardian. Enrollment occurred between January 2018 and September 2021; participants enrolled at the end of the study were followed up until September 2022. All participants were observed for 24 months, except those enrolled toward the end of the study period who were observed between 12 and 23 months. Periodic safety review was conducted by a 3-member data safety monitoring board.

### Intervention

Full details regarding the intervention are provided in the trial protocol^11^ and summarized here. The intervention (personalized transition support, access to a PN) was designed to overcome barriers and challenges experienced by participants by facilitating a coordinated entry into the adult system, to reduce ED/urgent care visits (primary outcome), and to increase the use of adult-oriented ambulatory primary and specialty care. There was 1 PN in each of Calgary and Edmonton at any one time during the study, and these individuals were employed by Alberta Health Services, the sole health authority providing universal access health care to residents of Alberta. Navigators had a minimum of a bachelor’s degree in social work and 5 years of clinical experience in health care. The navigators were each responsible for approximately one-half of the total enrolled patients (accruing patients over time); some participants completed the study while the recruitment was ongoing.

We developed the structured PN intervention with 4 distinct interrelated modules (Table 1)^15^ based on literature highlighting the need for each, our own pretrial qualitative findings,^16^ collaboration with content experts in transition models, and partners within Alberta Health Services and patient/parent advisory committees. We developed a 2-day training program for the PN to complete before the start of the trial. The training consisted of readings, case scenarios, and role plays.

**Table 1.** Description of Patient Navigator Interrelated Modules Used to Deliver the Intervention

Module topic	Description
Preparation for transfer of care	Completing needs, risk, and transition-readiness assessments using a structured approach with modified SSHADESS psychosocial assessment, creating a medical passport, helping establish relationships with primary care practitioners and appropriate specialty care clinicians, and enabling timely attendance at first adult clinic visit.
Patient navigator as health system broker	Assisting with data sharing between pediatric and adult service practitioners, working with patient and primary care providers to facilitate continuity of care, promoting communication, promoting collaboration and patient- and family-centered care between all health care professionals, and advocate with/for parent/family.
Assisting with social determinants of health	Assisting families with barriers related to social and economic determinants of health to reduce modifiable barriers to accessing ambulatory medical care after transfer.
Promoting self-management of medical conditions	Providing information and access to tools, educational resources, and peer support groups; tracking follow-up clinic visits, medication refills, and laboratory testing to flag nonadherence early and provide coaching to reduce barriers to adherence; and planning for medical and/or mental health crisis management.

Abbreviation: SSHADESS, strengths, school, home, activities, drugs and substance use, emotions eating and depression, sexuality, safety.^15^

Once a participant was randomized to the intervention group, the PN attempted to make contact within 7 days to schedule a face-to-face or telephone meeting to conduct an initial assessment. Using the information obtained from the initial assessment, the PN used an adaptive^10^ patient-centered approach that customized delivery of services based on the needs of the participant. Scheduled patient reviews occurred every 3 months in person or by telephone (with text messaging capability). Every contact with the participant was recorded using standardized forms, and notes were also copied into electronic medical records. Navigators were alerted to participants’ ED/urgent care visits by either the participants, parent/guardian (if enrolled), clinical practitioners or through electronic medical record alerts. The study overlapped with the onset of the COVID-19 pandemic. The navigators quickly moved to virtual methods of contact and initial assessment, as they had already been set up to contact patients in this manner before the pandemic.

### Usual-Care Group

Participants assigned to the usual-care group received usual care as available within their pediatric clinic, which was variable across clinics and specialties. The study team provided usual-care participants with quarterly newsletters regarding transition resources and self-management tools.

### Outcome Measures

The primary outcome was the rate of all-cause ED/urgent care visits during the observation period. Participants’ personal health numbers were used to link with health service utilization data obtained from the Canadian Institute for Health Information Discharge Abstract Database. All ED/urgent care visits attributed to participants were obtained from the National Ambulatory Care Reporting System and represent a visit to an acute care ED or urgent care center providing emergency services.^14,17^ Complete data were available for ED visits (irrespective of which ED they presented to within Alberta) due to the universal health care system.^14^

### Covariates

Age, sex at birth, race and ethnicity, immigrant status, primary diagnosis, and comorbid conditions including mental health conditions were collected from the participant at enrollment by self-report. Ethnicity and immigrant status were self-reported and used to assess for diversity of the study population. Patients self-reported the following races and ethnicities: Asian, Black, Indigenous, White, or other race or ethnicity. Comorbid mental health conditions were self-reported and listed in the data collection form as follows: depression, anxiety, obsessive mental health disorder, eating disorder, schizophrenia, posttraumatic stress disorder/trauma, bipolar disorder and other (participants responded yes or no in the baseline medical comorbidity form). Socioeconomic status, as indicated by the Pampalon Index,^18^ and rural vs urban residence location was derived from the home postal code based on 2016 Canadian census data.

### Randomization and Masking

Participants were randomly allocated to either the intervention or usual care in a 1:1 ratio using a computer-generated randomization sequence, with varying block sizes, stratified by primary clinic affiliation. Randomization allocation was executed using REDCap (Vanderbilt University) research software.^19^ Intervention assignment was not blinded from trial participants, family members, research assistants, or clinical teams. All AEAs and parent/guardian participants were blinded to the primary outcome and hypothesized effects of the study.

### Statistical Analysis

The baseline ED/urgent care visit rate observed within a diverse cohort of transitioning patients at the Alberta Children’s Hospital, as identified using available administrative data, was 51 per 100 person-years of follow-up, for age 18 years or older (based on internal data). Our team, composed of end users from various levels of health service delivery (pediatric and adult specialist care providers, emergency department physicians, health service delivery administrators, patient council), confirmed that a minimum clinically important difference between groups is a 20% (relative change) drop in ED visits. Based on the effect size observed in a prior study evaluating transition navigators’ impact on diabetic ketoacidosis admissions in patients with diabetes, we expected a 20% to 25% relative rate reduction in the intervention group compared with the control group. Based on the comparison between 2 rates, assuming an ED/urgent care visit rate of 40 per 100 person years (21% rate reduction) in the intervention group, with significance level of α = .05 and 80% power, a sample size of 300 per arm was determined. We projected an average follow-up of 2.04 years based on 24 months of recruitment and 36 months of maximum observation for outcomes. Loss to follow-up did not affect our ability to measure the primary outcome, as we used administrative data. The recruitment time was extended from 24 months to 42 months, in response to slow recruitment. We used multiple strategies to inform potential participants about the study, including creating attractive, youth-friendly posters, having a study champion in each clinic, and invitation letters. Recruitment was severely hampered by the COVID-19 pandemic. The study team decided to stop enrollment in September 2021 due to lack of funding to continue the study beyond a reasonable period.

All analyses were conducted according to intention to treat. Descriptive statistics, including frequencies, percentages, mean and SD, and median and IQR, were used to describe the demographic and clinical factors for intervention and usual-care cohorts. ED/urgent care visits per 100 person-years were calculated with 95% CIs. Zero-inflated negative binomial regression was used to calculate the incidence rate ratio (IRR) due to overdispersion in ED visit rates and the large proportion of participants with zero ED visits. The IRR with 95% CI was estimated for the intervention compared with the usual-care group, in multivariable zero-inflated negative binomial models while adjusting for age, sex, race and ethnicity, Pampalon Deprivation Index, rural/urban location, COVID-19 pandemic time period, and self-reported mental health comorbidities. To explore the effect of the COVID-19 pandemic on ED/urgent care visits, we grouped events observed before vs after the pandemic declaration (March 11, 2020). To test for effect modification, interaction terms (using the list of baseline variables and comorbidity collected in the study) were added to the model. Interaction terms were only retained if they were significant. A final model with relevant clinical covariates, and best model fit according to the Bayesian information criterion was selected. Follow-up years were calculated accordingly, excluding negative or zero follow-up years. Statistical significance was set at α = .05, all *P* values were 2-sided, and data were analyzed using SAS statistical software, version 9.4 (SAS Institute).^20^

## Results

### Participant Characteristics

A total of 541 participants were assessed for eligibility, of whom 206 were excluded, and 335 were randomized over a period of 45 months (Figure), 164 (49.0%) to the intervention arm and 171 (51.0%) to usual care. After 1 patient withdrew and requested complete data withdrawal, 334 participants (usual care: mean [SD] age, 17.8 [0.7] years; 99 female [57.9%]; 71 male [41.5%]; 1 missing [0.6%]; intervention: mean [SD] age, 17.7 [0.6] years; 79 female [48.5%]; 81 male [49.7%]; 3 other/prefer not to say [1.8%]) were included in the final data analysis. The intervention and usual-care groups had similar baseline demographic and clinical characteristics (Table 2). Among the participants, 131 (39.2%) resided in a rural location, and 126 (37.7%) had a self-reported mental health comorbidity during baseline assessment. Participants self-identified with the following races and ethnicities: 41 Asian (12.3%), 15 Black (4.5%), 30 Indigenous (9.0%), 230 White (68.9%), 12 other race or ethnic group (3.6%), and 6 missing data (1.8%). In the navigator arm, 14 patients withdrew from the intervention Figure). No patients actively withdrew from the usual-care arm. Two participants moved out of province during the study period (1 from each group); for these patients, the observation period was truncated on the move date. No patients died during the observation period. There were 126 participants (63 in the navigator group, and 63 in intervention group) who reported that they had at least 1 co-occurring mental health condition; 80 selected 2 or more, with anxiety being most reported (104 [82%]) followed by depression (70 [56%]). Thirteen participants (8 from usual care and 5 from navigator group) reported that the mental health condition was their primary disorder.

**Figure.**
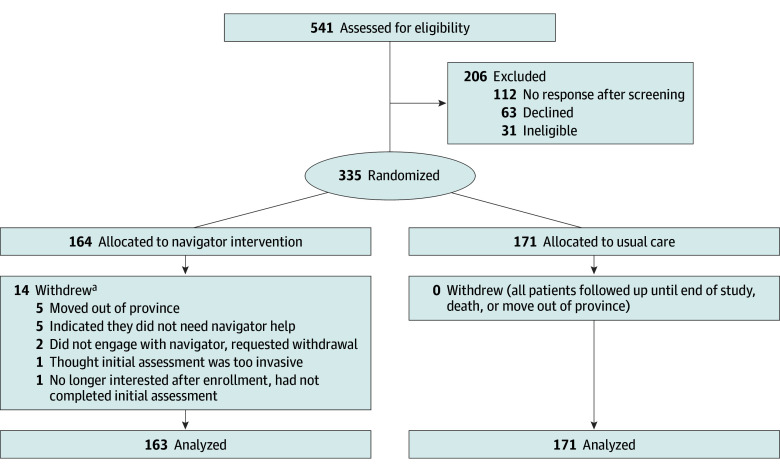
Consolidated Standards of Reporting Trials Diagram Showing Flow of Participants in the Trial ^a^One participant requested complete data withdrawal.

**Table 2.** Sociodemographic Characteristics of Participants at Baseline

Characteristic	No. of patients (%)	
	Usual care (n = 171)	Navigator (n = 163)
Age, mean (SD), y	17.8 (0.7)	17.7 (0.6)
Sex (self-reported)		
Female	99 (57.9)	79 (48.5)
Male	71 (41.5)	81 (49.7)
Other/prefer not to say	0 (0.0)	3 (1.8)
Missing	1 (0.6)	0 (0.0)
Ethnicity (self-reported)		
Asian	25 (14.6)	16 (9.8)
Black	7 (4.1)	8 (4.9)
Indigenous	18 (10.5)	12 (7.4)
White	113 (66.1)	117 (71.8)
Other	7 (4.1)	5 (3.1)
Missing	1 (0.6)	5 (3.1)
Primary clinic		
Genetic/metabolic	8 (4.7)	9 (5.5)
Neurological	31 (18.1)	30 (18.4)
Cardiovascular	20 (11.7)	20 (12.3)
Respiratory	7 (4.1)	5 (3.1)
Kidney/urinary tract	13 (7.6)	10 (6.1)
Gastrointestinal/liver	14 (8.2)	14 (8.6)
Hematology/immune deficiency	11 (6.4)	8 (4.9)
Rheumatology	31 (18.1)	32 (19.6)
Endocrine	19 (11.1)	20 (12.3)
Mental health	8 (4.7)	5 (3.1)
Developmental/sensory	8 (4.7)	8 (4.9)
Other/null	1 (0.6)	2 (1.2)
Pampalon Index, quintile		
1 (Least deprived)	35 (20.5)	26 (16.0)
2	43 (25.1)	39 (23.9)
3	30 (17.5)	39 (23.9)
4	40 (23.4)	24 (14.7)
5 (Most deprived)	23 (13.5)	35 (21.5)
Location		
Rural	66 (38.6)	65 (39.9)
Urban	105 (61.4)	98 (60.1)
Mental health condition (self-reported)		
Yes	63 (36.8)	63 (38.7)
No	108 (63.2)	100 (61.3)
Immigrant status (self-reported)		
Yes	15 (8.8)	19 (11.7)
No	156 (91.2)	141 (86.5)
Missing	0 (0.0)	3 (1.8)

### Frequency of Contact With Navigator

In total, 287 participants had access to the navigator for 24 months, and 47 had access less than 24 months. All participants had a minimum of 12 months access to the PN, unless they withdrew from the study. The range per participant of all types of contacts with the navigator was from 0 (due to withdrawal or nonresponse to contact request by navigator) to 61 over the course of the intervention period. These contacts included both scheduled (initiated by navigator as prescribed by study timeline) and unscheduled (initiated either by navigator or participant for routine or emergent needs) contacts. Navigators supported participants in a variety of ways and tailored their intervention to the specific needs of the participant. The types of tasks most frequently reported by the PN (according to fidelity checklists) were monitoring participant adherence to medical care, supporting appropriate engagement with health and mental health services, and coaching self-management skills and identifying strategies to promote adherence.

### Outcomes

Table 3 summarizes the observed ED visit rates per 100 person-years of observation by intervention vs control and by sociodemographic characteristics. The majority of these visits occurred in adult care due to the design of the study timeline. In the cohort, 149 participants (44.6%) had no visits recorded during observation. Overall, for the cohort, those with mental health conditions had significantly higher ED visit rates compared with those without (unadjusted IRR, 2.32; 95% CI, 1.54-3.49; *P* <.001). The period of observation before the COVID-19 pandemic was associated with a higher ED visit rate than following the onset of the pandemic (unadjusted IRR, 1.48; 95% CI, 1.09-2.01; *P* =.01).

**Table 3.** Crude Emergency Department Visit Rates by Sociodemographic Variables for Intervention and Control Groups

Characteristic	Intervention group				Control group			
	No. of participants	No. of events	Person-years	Rate (95% CI)^a^	No. of participants	No. of events	Person-years	Rate (95% CI)^a^
Age, y								
16-17	120	216	231.5	93.3 (81.7-106.6)	123	274	245.0	111.9 (99.4-125.9)
18-20	43	86	78.2	109.9 (89.0-135.8)	48	64	95.5	67.0 (52.5-85.6)
Sex								
Female	79	160	149.3	107.2 (91.8-125.1)	100	259	198.8	130.3 (115.3-147.1)
Male	81	139	155.8	89.2 (75.5-105.3)	71	83	141.6	58.6 (47.3-72.7)
Other/prefer not to say	3	3	4.6	65.3 (21.1-202.6)	NA	NA	NA	NA
Missing	NA	NA	NA	NA	1	2	2	100.0 (25.0-399.9)
Ethnicity								
Asian	16	13	31.8	40.9 (23.7-70.4)	25	59	49.4	119.4 (92.5-154.1)
Black	8	29	16	181.3 (126.0-260.8)	7	23	14	164.3 (109.2-247.2)
First Nation/Indigenous	12	53	24	220.8 (168.7-289.1)	18	46	36	127.8 (95.7-170.6)
White	117	196	218.9	89.5 (77.8-103.0)	114	201	227.3	88.4 (77.0-101.5)
Other	5	4	9.8	41.0 (15.4-109.1)	7	12	13.7	87.7 (49.8-154.3)
Missing	5	7	9.2	75.8 (36.2-159.1)	1	3	2	150.0 (48.4-465.1)
SES Pampalon Index, quintile								
1 (Least deprived)	26	29	49.7	58.3 (40.5-83.9)	36	95	71.7	132.5 (108.4-162.1)
2	39	52	70.4	73.9 (56.3-97.0)	43	60	85.1	70.5 (54.7-90.8)
3	39	61	75.9	80.4 (62.6-103.3)	30	37	59.9	61.8 (44.8-85.3)
4	24	28	44.3	63.3 (43.7-91.6)	40	108	79.7	135.4 (112.2-163.6)
5 (Most deprived)	35	132	69.6	189.7 (160.0-225.0)	23	44	46	95.7 (71.2-128.5)
Location of residence								
Rural	65	132	125.4	105.3 (88.8-124.8)	67	179	133.8	133.7 (115.5-154.8)
Urban	98	170	184.3	92.2 (79.3-107.2)	105	165	208.6	79.1 (67.9-92.1)
Participant immigrant status								
No	141	267	267.3	99.9 (88.6-112.6)	157	320	312.7	102.3 (91.7-114.2)
Yes	19	30	36.4	82.3 (57.6-117.8)	15	24	29.7	80.8 (54.2-120.6)
No response	3	5	6	83.3 (34.7-200.2)	NA	NA	NA	NA
Participant mental health comorbidity								
No	100	153	192.5	79.5 (67.8-93.1)	108	107	215.1	49.7 (41.2-60.1)
Yes	63	149	117.3	127.1 (108.2-149.2)	64	237	127.3	186.1 (163.9-211.4)
COVID-19 pandemic								
Prepandemic (before March 11, 2020)	137	165	132.6	124.4 (106.8-145.0)	143	158	141.2	111.9 (95.7-130.7)
During the pandemic (after March 11, 2020)	157	137	179.9	76.1 (64.4-90.0)	169	186	202.8	91.7 (79.5-105.9)

Abbreviations: NA, not applicable; SES, socioeconomic status.

^a^ Per 100 person-years.

The final model comparing ED visit rates in the intervention and control group was adjusted for age, sex, race and ethnicity, Pampalon Deprivation Index, mental health comorbidity status, the COVID-19 pandemic, and residence location. The relationship between the intervention and ED visit rate was modified by participant’s mental health status; no other interaction terms considered were significant (ie, sex × intervention, residence location × intervention). Among those with mental health comorbidity, we observed approximately 25% lower ED visit rates in the navigator group compared with the control group, however, this association was not statistically significant (adjusted IRR, 0.75; 95% CI, 0.47-1.19; *P* = .22). Among those without a mental health comorbidity, we observed a higher ED visit rate in the navigator group compared with the control group (adjusted IRR, 1.45, 95% CI 0.95-2.20; *P* = .08). This association was also not statistically significant.

## Discussion

Provision of a personalized patient navigator intervention, embedded within routine care of a universal health care system, was not associated with a decrease in ED visits. Nonetheless, approximately 25% reduction in ED visits was observed among those with a self-reported mental health comorbidity and access to the navigator. The magnitude of this reduction may suggest a clinically important benefit of the intervention to this subgroup of patients.

To our knowledge, this was the first RCT to assess the impact of a PN intervention among a diverse group of AEAs transitioning from pediatric to adult care. This study provides an example of how a health service intervention can be embedded into care of those needing navigator services such as those with medical complexity, youth, or seniors. Manwani et al^9^ conducted an observational study analyzing the impact of a navigator intervention for AEAs with sickle cell disease undergoing transition. This study reported a significant improvement in transition readiness scores, disease knowledge scales, and confidence in pain and disease management.^9^ Allemang et al^10^ conducted a retrospective observational study examining the impact of a PN intervention for AEAs with hemoglobinopathies. This study reported improved treatment adherence and reduced loss to follow-up (*P* < .05). However, the frequency of hospitalization was not different before and after the PN intervention.^10^ Published studies describing PN services are mostly single-center and single-disease cohort studies, with nonrandomized designs, thus, limiting generalizability to other health jurisdictions and disease populations.^4,7,9,10^ The PN service in our study was accessible to a broad range of AEAs with chronic health and mental health conditions, and the service was embedded in routine health care, improving generalizability.

AEA with self-reported co-occurring mental health conditions had higher ED visit rates compared with those without co-occurring mental health conditions. Similar trends have been noted in other studies. Chiu et al^21^ reported that in Ontario, Canada, mental health–related ED visit rates increased by 89.1% between 2006 to 2017 among AEAs. Poonai et al^22^ reported that mental health–related ED visit rates and hospital admissions, particularly related to suicidal ideation, self-poisoning, and self-harm, continued to increase during the first 2 years of the COVID-19 pandemic among adolescent females in Canada. Villas-Boas et al^23^ observed that after the pandemic, the demand for mental health care among AEAs exhibited a quicker rebound to prepandemic levels compared with overall ED visits, highlighting a distinctive trend in health care–seeking behavior. These published studies draw their cohorts from the general population; however, our study confirmed that those with a chronic condition and a self-reported mental health comorbidity used the ED more. Observed IRRs were 2.3-fold higher compared with those without a mental health comorbidity. Importantly, we found that the intervention group experienced lower ED visit rates among those with a mental health comorbidity. The personalized navigation service was poised to deliver care virtually and using telephone and text messages even before the onset of the pandemic. The magnitude of the difference in effect estimates, although nonsignificant, may suggest a clinical benefit of providing the navigator to those with mental health comorbidity.

Although the pandemic severely affected recruitment rates for this trial, the mode of delivery for the intervention did not change substantially. The navigators continued to use cell phones and virtual means to contact AEAs, complete assessments, and provided similar advice before vs after pandemic onset. This could be one potential explanation why we did not observe effect modification by pandemic onset within the adjusted model.

We observed that Asian, Black, and Indigenous participants were more likely to visit the ED. This raises important questions about barriers to primary health care or cultural differences in accessing care^24^ and disparities in access to health care information for new immigrant communities to potentially avoid an ED visit.^25^ Further, our results also suggest the need to evaluate pathways through which structural racism may affect health, including economic disparities, and timely access to health care and treatments.^26^ Consistent with other studies, males exhibited lower ED visit rates than females. Rural dwellers were also more likely to use the ED, but this may be partially explained by reliance on hospital-based services for basic care in some rural and remote regions.^27^

### Limitations

Our study has limitations. Patient-reported outcome measures such as pediatric quality of life were not included, though qualitative data on the intervention will be reported elsewhere. We were unable to categorize ED visits as avoidable or unavoidable and, therefore, chose to use all-cause ED visits as our primary outcome. Although we hypothesized that ED visits are an outcome of poor transfer, and possibly due to lack of appropriate engagement with health care services including primary care during the transition period, we could not assert that this was the only mediating factor due to the heterogeneity of participants enrolled in the study. The pandemic severely affected recruitment and, thus, we were unable to reach the target sample size. We did not capture the severity of mental health conditions self-reported by participants in this study. The majority of participants were White, despite robust recruitment efforts to increase diversity, which limits generalizability to racialized populations. Our study also did not have process measures defined to measure the impact of the 4 modular domains of the navigator intervention.

## Conclusions

Results of this parallel-group randomized clinical trial demonstrate that the comprehensive PN intervention did not impact the rate of ED visits among a large sample of AEAs across a large geographic jurisdiction during the transition to adult care. There may be a benefit to those individuals with mental health comorbidity. The study did not accrue sufficient sample size to demonstrate a significant difference between groups should it exist.

## Notes

Supplement 1.
Trial Protocol.

Supplement 2.
Data Sharing Statement.
