# Supplementary material for: Effectiveness of Patient Navigation During Transition to Adult Care: A Randomized Clinical Trial
Source: JAMA Pediatr. 2025 Feb 10;179(4):375–82. doi: 10.1001/jamapediatrics.2024.6192 (PMC11811865; doi:10.1001/jamapediatrics.2024.6192)
Supplement: Supplement 1. — Trial Protocol. [file jamapediatr-e246192-s001.pdf]

OVERVIEW

Transition to adult care is a challenging and complex time for youth with chronic health conditions.[1] Providing uninterrupted coordinated care during this period is critically important to maintain health in this vulnerable population.[2,3] The Transition Navigator Trial is a pragmatic randomized controlled trial (RCT) of a patient navigator service versus usual care, aimed at adolescents aged 16-21 years living with a chronic health condition in Alberta who are transferring to adult healthcare. This mixed-methods (quantitative and qualitative) study will address modifiable barriers to successful transfer of care, and evaluate cost savings attributable to the intervention (Figure 1). This study addresses one of the top priorities for patients and families, pediatric and adult health care providers, and policy makers in Canada.[4,5]

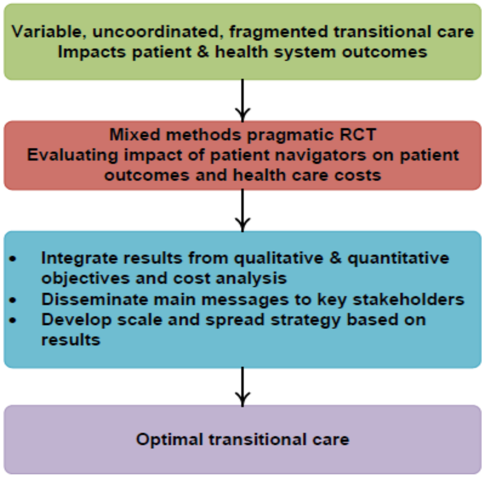

Figure 1: Transition Navigator Trial Overview

SIGNIFICANCE AND IMPACT OF RESEARCH

Approximately 15-20% of adolescents in North America live with a chronic health condition, and the majority (>90%) will require transfer from pediatric to adult oriented services.[1,2] Many patients and families are unprepared for transfer,[6-8] struggle to adapt to adult health care settings,[9,10] and report difficulties accessing needed services to manage their health conditions into adulthood.[11] Similarly, adult care providers feel unprepared to care for transferred pediatric patients,[12,13] and lack necessary resources and time to manage the complexity and diversity of their psychosocial and medical needs.[14-16] As a result, transferred patients experience difficulties navigating an unfamiliar adult health care system,[10] and some experience significant and irreversible health deterioration and increase in co-morbidities.[3,6,17,18]

Sub-optimal transition to adult care leads to poor compliance with ambulatory care management, health deterioration, and increased use of costly emergent health services.[19,20] Young adults with diabetes experience 20% increased admission rates for diabetic ketoacidosis[21] and those with kidney transplant experience two fold risk of transplant failure immediately after transfer of care.[22,23] Fifty percent of transferred congenital heart disease patients fail to attend an adult clinic within 3 years.[24,25] Among those with a lapse in care, 23% will need a cardiac catheter or surgical intervention within 6 months of returning to care and are 4 times more likely to require urgent intervention compared to those who do not lapse in care.[26] High acuity emergency room (ER) visits increased after transfer among young adults with chronic conditions in Ontario.[27] In our preliminary work using health administrative data available in Calgary, we found that among 637 pediatric patients identified as 'transitioning to adult care', there was an 18% relative increase in ER visits and 50% relative increase in intensive care unit length of stay from age 18 to age 19. These data highlight the need to provide coordinated, uninterrupted and patient-oriented **transition** programs to support youth and young adults with chronic health needs transferring to adult health care.[28]

Patient navigators are a promising, but unproven intervention to facilitate planned transitions to adult care. Clinical practice guidelines[2,29] highlight the importance of coordination, collaboration and communication between adult and pediatric care teams during transition, measuring transition readiness using validated tools (e.g. Transition Readiness Assessment Questionnaire)[30], and providing access to a

patient navigator to coordinate care.[31,32] Patient navigators address barriers to care for transitioning patients by providing patient-oriented health system navigation, tailored education and tools to promote self-management (e.g. a portable medical summary[2]), and psychosocial support.[33,34] The implementation of a patient navigator service for type 1 diabetics in Manitoba reduced hospitalizations for diabetic ketoacidosis by 37% and improved markers of diabetes control.[19] Other studies in patient cohorts with diabetes,[35-37] sickle cell disease,[38] and rheumatic disease[39] patients have reported a shift in health care utilization from unnecessary acute and emergency care to more appropriate, less costly, preventative ambulatory care. Liver transplant patients with access to navigators demonstrated improved adherence with immune suppression drugs[33] and kidney transplant patients with access to a transition clinic maintained graft function after transfer.[20] Published studies to date are mostly single centre, single disease cohort studies, with non-randomized designs, thus, limiting generalizability to other health jurisdictions.[40] No study to date has evaluated the benefit of a patient navigator service to improve patient or health system outcomes, when implemented across the health system in multiple chronic disease clinics, using a pragmatic randomized control design.

Therefore, we will conduct a RCT using mixed methods[41] to address the following objectives:

1. Evaluate the impact of a patient navigator intervention for adolescents and young adults with chronic health conditions (age 16-21) transferring to adult oriented services, on ER visits (primary outcome), other health care utilization measures, and transition readiness assessment scores (secondary outcomes).
2. Develop an in-depth understanding of adolescents, young adults and their families' perceptions about the transition process to adult health services within Alberta, and their reflections on a patient navigator service to address modifiable barriers to adult oriented care.
3. Create a comprehensive cost model to determine the net health care cost savings attributable to the patient navigator intervention.

We hypothesize that the patient navigator intervention will reduce all-cause ER/urgent care visit rates among transitioning young adults, and generate cost savings for the health system.

Pilot Work: We developed this study based on pilot work to innovate transition to adult services in Alberta. The Stollery Children's Hospital (SCH) Pediatric Diabetes Center employs a transition care navigator model to provide support to adolescents and young adults transferring to adult care. The program and its clinical lead (K. Johnston) have transferred 139 young adults to adult diabetes using this service since 2014. The Alberta Children's Hospital (ACH) in Calgary has an established transition program (Well on Your Way, staffed by D. Thul) and provides case navigation for selected medically complex patients. Dr. Mackie (co-Principal Investigator) of the SCH Cardiology program has successfully completed 2 RCTs evaluating the impact of nurse-led interventions to improve knowledge and self-management skills adolescents with congenital heart disease transferring to adult care.[42,43] Results of these studies led nursing staff to stay in contact with cardiology patients by text messaging or emailing (based on patient preference) during transition, and this has allowed for ongoing patient assistance and education after transfer of care.[44]

Impact: This pragmatic RCT directly addresses the call to action of the 2016 Canadian Association of Pediatric Health Centres Guideline for the Transition from Pediatric to Adult Health Care[29]: "next generation of research [in transition to adult care needs to address limitations of prior studies including], small sample sizes, non-generalizability due to diagnosis-specific studies, inclusion of youth with medical complexity, and limited number of RCTs." Further, this study addresses one of the

top priorities set by the Maternal Newborn Child Youth Strategic Clinical Network within Alberta Health Services (AHS)[29] - "to develop a coordinated approach to guide the health care journey of the medically complex child." Patients, clinicians and senior AHS policy makers in Calgary and Edmonton have worked together since 2012 to develop this proposal, due to a **critical** need to improve transition for highly vulnerable youth with chronic health needs. Alberta is uniquely positioned to conduct a study of this scope and scale due to its single integrated provincial health care system. The results will have the **potential to change health service delivery across Alberta**, and **provide urgently needed data to improve health, care delivery and the experience of all young adults transitioning from pediatric to adult care**. Therefore, this study is directly relevant to the mandate of the Canadian Institutes of Health Research, "the creation of new knowledge and its translation into improved health..., [and] more effective health services" for all Canadians.

APPROACHES AND METHODS

Design: RCT with a mixed-methods (quantitative and qualitative) design (Figure 2).[41] The intervention we are testing requires exploration of contextual (patient/family/local health care/community) variables that may influence patients' experiences and implementation of the intervention across multiple clinics and locations.

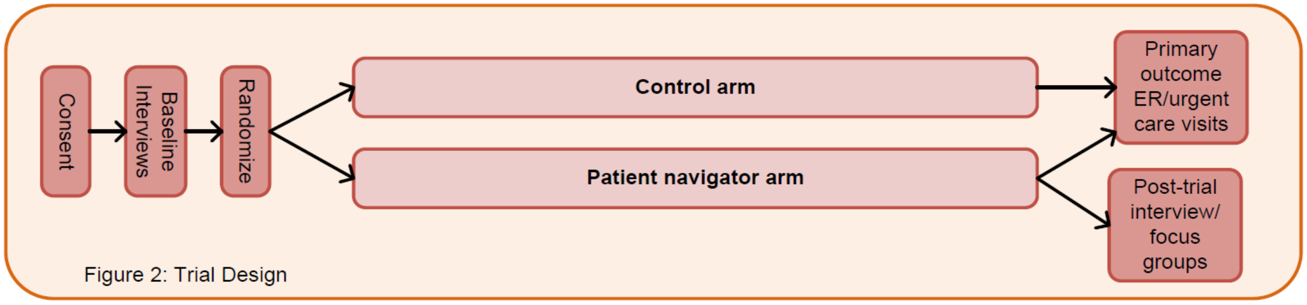

Figure 2: Trial Design

<u>Objective 1 – Randomized controlled trial of a patient navigator service</u>

Setting, clinic selection and identification of patients: Eligible participants will be identified from 30 pediatric specialty clinics at two acute care hospitals (Alberta Children's Hospital [ACH] and Stollery Children's Hospital [SCH]), and Glenrose Rehabilitation Hospital (GRH). The study coordinator will screen patient charts to determine eligibility, pass on names of eligible patients to the clinic staff, and clinic staff will approach patients about the study. If the patient is interested, the study coordinator will speak to them either in-person at their appointment, or virtually if the patient prefers, and completes a 'consent to contact' form. These hospitals are responsible for *all* tertiary pediatric health care in Alberta, thus providing an opportunity to recruit from a population based sample. Participants will have chronic health conditions in these broad categories: diabetes, endocrinology, gastroenterology, neurology, neurodevelopmental, rheumatology, nephrology, cardiology, hematology, respiratory, metabolic/genetics, autism and fetal alcohol spectrum disorder.
The clinics were selected after extensive stakeholder input and these patient groups have high potential for adverse outcomes if transitions are not managed optimally.

Inclusion criteria: All patients within the selected clinics, between the ages 16 – 21 years, with chronic medical conditions (defined as conditions which are >3 months in duration and/or lifelong with multiple morbidities and/or multi-organ/system manifestations or conditions which typically affect a single organ/system),[27,45] who are expected to be transferred to adult oriented specialty follow-up care will be screened for inclusion into the study. A single definition cannot describe all youth with complex care needs; therefore, we have chosen broad and pragmatic inclusion criteria to be consistent with recommendations in the literature.[46] The last planned pediatric visit should be within 6 months after

assessment of eligibility. Date of transfer to adult care will be defined as the final pediatric clinic visit. Potentially eligible patients will be identified by reviewing daily appointment lists ofparticipating

clinics using electronic appointment schedulers (eClinibase™) available at each site. We chose ages 16-21 for inclusion into the trial to be consistent with known transfer practices within the participating clinics/hospitals, and to capture both younger and older age transitions, potentially reflecting more challenging patients. Inclusion of a broad age range will reduce selection bias and increase external validity.

Exclusion criteria: Patients will be excluded if: 1) they are enrolled in another transition related study; 2) if transfer will not occur during the time interval for the trial; 3) the participant or parent/guardian cannot consent in English; or 4) they plan to move out of Alberta during the study period.

Randomization: The unit of randomization will be the patient, and each participant will be assigned to either the navigator service or control group for the duration of the study. Patients or clinic staff cannot select themselves into the intervention or control groups. Participants' consents will describe that the study is evaluating navigational support during transition to adult care, without providing details about hypotheses or the primary outcome. We expect that the study design will result in similar distribution of potential confounders (e.g., differences between sites and clinics) between the intervention and control groups. Some patients are followed in multiple clinics and at times in both Edmonton and Calgary, so cluster randomization by clinic will not be feasible.

Intervention: The intervention will be a patient navigator service. The navigator will address barriers and challenges experienced by transferring patients,[3,47] by facilitating a coordinated entry into the adult system and successful attachment to adult care.[47] The navigators will first schedule a face-to-face meeting for participants randomized to the intervention group. During this meeting the navigator will: 1) complete a needs assessment based on history provided by the patient, parent (if appropriate), 2) have the participant complete a baseline Transition Readiness Assessment Questionnaire (TRAQ);[30] 3) confirm that the pediatric clinical team has helped the patient create a medical passport using an online tool (www.sickkids.ca/myhealthpassport/) with key information needed to assist physicians in an emergency situation. Using this information, the navigator will create a patient-centred transition plan, in collaboration with patient, family, pediatric and adult providers, and community partners.

After the introductory visit, the navigators will assist patients with: 1) accessing primary care and/or specialty care as appropriate to address specific medical/mental health needs; 2) enable timely attendance at first adult clinic visit; 3) support adherence to follow-up clinic visits and health maintenance including completing required periodic laboratory tests and obtaining prescription refills; 4) assist with financial and insurance paper work; and 5) promote self-management by providing tools and educational resources and informing the participant of available peer-support groups as appropriate. Navigators will use electronic technology (cell phone, email, text message, fax, telehealth) to maintain contact and provide support for both urban and rural dwelling participants. The needs of each patient will vary from participant to participant and hence, it will be **patient-centred** and **patient-specific**.[47] There will be no *a priori* limits on the frequency of contact between the navigator and participant. The navigators will record every contact and nature of assistance provided using standardized encounter forms.

The duration of intervention will be a minimum of 12 months from enrolment (within 6 months of last planned visit to pediatric clinic) to a maximum of 24 months after transfer (measured from the date of the last pediatric visit). There will be one navigator for each of Edmonton and Calgary, who will have a minimum of Licensed Practical Nurse qualifications, and experience working with adolescents and/or young adults. The intervention group will also receive usual care as described below.

Control group: Patients assigned to the control group will receive usual care as available within adult and pediatric clinics and the health region; the control group is *not* a 'no intervention' group. These may include attendance at transition clinics, and access to available transition websites, self-management tools, and workshops. The usual care group will receive semi-annual newsletters from the research team regarding transition resources available in routine care. Significant variation in transitional care is expected for this group within and across sites (based on our prior stakeholder engagement work). We will use results of a detailed environmental scan (being conducted by Transition to Adult Care Committee of the Maternal Newborn Child Youth Strategic Clinical Network within AHS) of transition interventions in participating clinics, to determine if elements of the intervention are being offered by participating clinics. This heterogeneous control group will enhance study generalizability to other health care jurisdictions.

Blinding: Due to the nature of the intervention, treatment assignment cannot be blinded to the participant. Allocation will be concealed to the clinical teams at time of randomization. The statistician on our team (Nettel-Aguirre) will create the randomization sequence. The allocation for consented patients will be revealed to research staff, using automated web-based forms using REDCap database program (www.projectredcap.org). The research staff will then inform the navigator of a new patient assignment. Clinical staff may be un-blinded if/when the navigator contacts the clinical teams. All patient/family participants will be blinded to the primary outcome (ER/urgent care visit) and hypothesized effects of the study. The navigators will also be blinded to the primary hypothesis. Full details of the navigator intervention will not be available to clinic staff/participants to minimize contamination of the intervention to the control group. Consent forms will inform participants that we are studying how to best facilitate care of adolescents and young adults during the transition.

Standardized assessments: All included patients will complete the following standardized assessments at 0, 6, 18 and 24 months: Beck Depression Inventory, Beck Anxiety Inventory, Mind the Gap (Youth), and Adolescent Alcohol Drug Involvement Scale.

Lived experience journaling: All included patients will be invited to journal their experiences using an online REDCap form accessible to patients. These journal entries will be collated and evaluated using free text qualitative analysis to elicit common themes and experiences.

Caregivers/guardians: We will also obtain informed consent from caregivers/guardians to obtain demographic information and invite them to completed standardized assessments (Mind the Gap (Caregiver version), Beck Depression Inventory, Beck Anxiety Inventory).

Timeline: We will conduct the following preparatory work for this trial over the next 3-6 months while we await funding: 1) preparation of sites/clinics to launch study; 2) drafting of case report forms; 3) ethics applications to University of Calgary and University of Alberta; 4) developing standardized training modules for navigator service providers; 5) hiring patient navigators in collaboration with AHS leadership. Funds for these activities will come from existing funds held by the Principal Investigator (Samuel), cash support from AHS (see letters by Morrison and Westerlund) to hire navigators, and in kind support from AHS. Therefore, we will be ready to recruit the first patient within 6 months after start of funding and will continue recruitment for 24 months (month 30 of study). All enrolled patients will be observed for at least 12 months or at most 36 months (until month 42 of study). Maximum time period of navigator support is 24 months. See Figure 3 below.

Year 1		Year 2		Year 3		Year 4	
0-6 months	6-12 months	12-18 months	18-24 months	24-30 months	30 - 36 months	36-42 months	42-48 months
Preparation	Patient Recruitment						

	**Patient navigation and observation for outcomes in both groups**	
	**Baseline pre-randomization qualitative interviews**	**Post-trial qualitative interviews/focus groups**
		**Data Analysis/Dissemination of Results**

Outcomes: The primary outcome is rate of all-cause ER and urgent care visits during the observation period. Patients, providers, and policy makers on our team considered ER/urgent care visits to be relevant and measureable in all clinical groups being studied for impact of gaps in transitional care.

Secondary outcomes are: 1) health care utilization measures (frequency of in-patient admissions, ICU admissions, primary care and specialty ambulatory care visits, and ER visits/hospital admissions for ambulatory care sensitive conditions, defined as avoidable visits/hospitalizations with appropriate ambulatory care,[48] and length of hospital stay); 2) TRAQ score 12 months post-randomization (see below for details).

Data sources: In addition to detailed case report forms to document patient baseline characteristics, and navigator contact with patients and families, quantitative study data will come from two sources.

Administrative data: We have access to all the data needed to do this study, through the Clinical Analytics Team of AHS (see support letter from A. Ryan). AHS is the custodian of all Alberta Health data including outpatient/inpatient health care encounters and emergency visits, intensive care unit admissions and ambulatory care visits (for >99% of population). We will obtain consent from participants enrolled in the study to use their personal health numbers to link with health service utilization data. As we are using secondary data sources to determine primary outcome, outcome assessments will be blinded to allocation of study of groups. Hospital admissions for ambulatory care sensitive conditions will be determined using the Canadian Institute for Health Information indicator libraries available online (www.cihi.ca) and using established methodology for chronic conditions for which these are defined.[48]

TRAQ: The TRAQ is the most rigorous transition readiness scale for adolescents. Sawicki et al. identified behaviors relevant to transition (self-management, self-advocacy, activities of daily living, use of resources), tested item reliability and validity, and then field tested the items with 194 youth with special healthcare needs. Scores range from 1 (low) to 5 (optimal). The questionnaire consists of 29 items, at grade 5.7 reading level, and takes ~5 minutes to complete.[30] Participants will complete the TRAQ online at enrolment (baseline), 6 and 12 months later. These time points were chosen because a) Mackie et al. demonstrated higher TRAQ self-management scores in a transition intervention group by 6 months[42] and b) participants enrolling later in the study will have exposure to the intervention for only 12 months.

Data analysis: We will use intention to treat analysis. We will use Poisson regression to compare rates of ER/urgent care visits between the navigator service and control groups. Important demographic and medical characteristics that could be potential confounders or independent predictors (e.g. age, disease type, socioeconomic status, location of residence, co-morbidity, ethnicity etc.) will be decided and collected *a priori*, and used for adjusting the Poisson model. All secondary outcomes will be analyzed using descriptive statistical methods and by key demographic variables. Using within person change in TRAQ scores as the variable of interest, we will compare between intervention and control groups via a 2 independent sample t-test.

Sample size: Our sample size calculation is based on the primary outcome ER/urgent care visit rate during the period of observation. The baseline ER/urgent care visit rate observed within a diverse cohort of transitioning patients in Calgary, as identified using available administrative data, is 51 per 100 person years of follow-up, for age ≥18 years. Our team, composed of stakeholders from various levels of health service delivery, confirmed that a minimum clinically important difference between groups is 20%. Based on this and effect size seen in a prior study evaluating transition navigators' impact on diabetic ketoacidosis admissions in diabetic patients,[36] we expect a 20-25% relative rate reduction in the intervention group compared to the control group. Assuming an ER/urgent care visit rate of 40 per 100 person years (21% rate reduction) in the intervention group, with significance level of alpha = 0.05 and 80% power, with an average follow-up of 2.04 years based on 24 months of

recruitment and 36 months of maximum observation for outcomes, the needed sample size in each arm is 300 (total sample 600, average 25 patients per month).

Feasibility of recruitment: Using administrative data, we estimate that there are approximately 600 patients between 16-18 years of age receiving care at ACH and a similar number at SCH. GRH has approximately 250 patients between the age 16-18 years. Estimated consent rate is 79% (based on our experience with transition trials).[42,43] We expect to recruit approximately 1 patient per month per participating clinic in each hospital site to reach target sample size. Site champions have been identified at all participating clinics to enable maximal recruitment. We estimate a case-load of 140-150 patients per patient navigator (one each in Edmonton and Calgary), based on feasibility assessments provided by diabetes navigator who performs similar work in Edmonton.

We have created a study introduction video to be used as a tool in the consent process, for example when a patient is interested in a study, but wants to discuss it with their parents before enrolling, we can share the video with them to help their conversation.

Recruitment via social media: We will also recruit potential participants through social media channels. We will set up a Facebook page for The Transition Navigator Trial, and post recruitment messages, approved by ethics and in-line with our existing recruitment materials. These posts will be boosted on Facebook and Instagram through paid advertisement to reach two key target audiences: youth who live in Alberta with chronic health conditions, and their parents.

Our recruitment materials will encourage readers who are interested in the trial to click through to a REDCap data collection form. The REDCap form will provide some background information about the trial. Respondents will have the opportunity to answer a few screening questions and leave their contact information for a research coordinator to follow-up with them about eligibility to join the trial. They will also have the option to download the study information letter.

> Do you/does your child go to appointments at the Stollery Children's Hospital, The Glenrose Rehabilitation Hospital or Alberta Children's Hospital? Y/N
> Are you/is your child between the ages of 16-21? Y/N
> Do you/does your child live in Alberta? Y/N

Name:
Phone Number:
Email address:
You may be eligible for the Transition Navigator Trial! A research coordinator will follow-up with you.

We will also ask community partners to share our social media images on their channels.

Some social media posts will target health care providers who may be able to refer eligible patients. They can refer patients by asking their patients to contact the TNT team (self-referral) or by completing a consent to contact form, or obtaining verbal consent to contact and conveying the information to our study team via AHS email or phone call.

Ethics and consent: We are studying both adolescents/young adults and their family members, and consent will be sought from all participants. For patients who are still minors, informed assent will be obtained where appropriate. When the patient is considered a mature minor (after a capacity assessment by responsible physician) or at age 18, we will obtain consent from the patient for participation as appropriate. If a patient is consenting for him/herself, then consent forms will ask patient participants

for permission to contact their parents/guardians as needed to facilitate care, and also for permission to disclose medical information to parents/guardians. Should the participant deny parent involvement in the study, parents will not be contacted during the intervention nor will health information be provided to the parent. We will request consent from all participants to allow linkage to their health administrative data using personal health numbers. Ethics approvals for this study will be sought from both the University of Calgary (Conjoint Health Research Ethics Board) and the Health Research Ethics Board at the University of Alberta.

Where obtaining written consent is not feasible, for example when patients are not being seen in the hospital by the research coordinator (due to COVID-19 pandemic), and patients do not have internet access at home for electronic consent, we will instead do a verbal consent and assent process.

Trial management: The trial protocol will be registered at clinicaltrials.gov and published in a peer reviewed journal. We will adhere to best practice guidelines according to the SPIRIT Checklist for RCTs.[49-51] The trial will be managed by a study coordinator in Edmonton, research assistant and data analyst in Calgary, with direction from a trial steering committee (composed of investigators, AHS leadership, patient representatives). We will be monitoring for potential adverse events in both study groups, however, the intervention is considered to be of minimal risk. No interim analysis is planned.

Objective 2 – Perceptions of transition and reflections on patient navigators

Design and methods: A qualitative study methodology [52] will be embedded within the quantitative RCT design. This methodology is recommended when conducting health service research with the aim of designing, implementing and evaluating an intervention [52] and when there are contextual variables that may affect both the process and outcome of a study, and inform fidelity and sustainability of the intervention in real health care settings.[53] Participants (youth and families) will be invited to participate in a 30-minute semi-structured telephone interview before randomization. This is to ensure that their responses are not influenced by the knowledge of their assignment to either the experimental or control group. Each participant (youth and/or parent) will describe and discuss their perceptions of how contextual (economic, legal, availability and accessibility of services) and individual factors (ethnicity, race, gender, age, type of diagnoses, family variables) will influence their ***upcoming*** transition and transfer experience from pediatric to adult services. Baseline interviews will not focus on the intervention. Fifty participants (youth and families) assigned to the intervention arm will be invited to participate in a second 30-minute semi-structured telephone interview within two weeks of the end of the trial, to reflect on the navigator service. Participants will also be asked about their experience with transfer of care during the covid-19 pandemic, and if/how this impacted their access to care. Key stakeholders (clinicians/policy makers) involved in the trial will also be invited to participate in focus groups to discuss perceptions

(process and implementation) of the navigator service; however, they will not be informed of the quantitative study results at this stage.

Sampling: Purposive sampling will be used to ensure that participants are selected based on maximum diversity for type and duration of illness and other salient demographics including ethnicity, race, gender, sexual orientation and socio-economic factors. We will invite 50 participants at the end of the navigator intervention (10 youth &10 parents at baseline; 15 youth & 15 parents) to participate in 30-minute telephone interviews. The final sample size will depend on the achievement of data saturation, and will also be determined by purposive sampling to facilitate maximum variation in demographics. Service providers (front line clinicians, policy makers) at three sites involved in the intervention arm will be invited to participate in a focus group (one for each participating hospital).

Data analysis: All interviews and focus groups will be audio-taped and transcribed verbatim. Thematic analysis will be used to extrapolate and systematically analyze patterns in the data generated by the qualitative interviews. We will closely adhere to the steps delineated by Braun and Clarke[54] for conducting thematic analysis. We will use Krueger and Casey's (2014)[55] constant comparative method of analysis to analyze the focus group data. This method involves "cutting, sorting, and arranging through comparing and contrasting." The coding process consists of grouping similar concepts and ideas, while identifying themes and categorizing results.[55] The research team will engage in established steps to increase the validity, credibility, transferability, and dependability of findings by adhering to guidelines for publication of qualitative research studies. [56,57]

Objective 3 – Cost Analysis

Design and Methods: We hypothesize that a patient navigator service will lead to reduction in emergency room utilization and hospital admissions (including intensive care unit admissions) by improving timely access to appropriate ambulatory care services to transitioning young adults. We expect a shift in health care utilization to more appropriate, less costly, preventative and health maintenance ambulatory care during the study period. We will prospectively capture the cost of the navigator intervention using microcosting methods[58] (identification, measurement, and valuation) that include one time and ongoing costs (development of materials, capital costs, wage rates for navigators, number of patients in caseload). The cost of this intervention per patient served will be calculated. Using linkage to high quality administrative datasets (from AHS Clinical Analytics data repository) that capture both the occurrence and cost of health care on a patient level basis (emergency room visits, hospital admissions including intensive care unit admissions, non-emergent ambulatory care visits), physician billing, and robust costing methods (Canadian Institute for Health Information Case Mix Group; Ambulatory Care case costing), we will determine and compare costs between groups with and without intervention using similar methodology as the primary outcome. The analysis will compare total costs as well as cost by category (in-patient, ER, non-emergent ambulatory care, physician claims) between both groups. Distributions of cost are often skewed and will be normalized, if necessary, by using log-transformations. Unadjusted analyses of costs will be performed using ANOVA; if cost cannot be normalized then nonparametric (e.g., Kruskal-Wallis) tests will be used. If we are required to use log-transformation of costs, we will use Duan's smearing estimator to calculate appropriate error estimates when the data are re-transformed from the logarithmic scale.[59] Patients will be censored at end of study to account for variable length of observation. A comprehensive cost model will be constructed to accurately determine the net health care cost savings attributable to the patient navigator intervention that incorporate the cost of the navigator intervention, cost savings from averted ER and hospitalizations (or reduction in intensity such as ICU and length of stay), and potential increased cost

of ambulatory clinics. Secondary analyses will focus on only ambulatory care sensitive ER and hospital admissions between groups.

Integration of qualitative and quantitative findings

The qualitative data will be used to explain the quantitative findings (differences in ER/urgent care visit rates between navigator and control groups), and put them in context. The research team and relevant stakeholders will develop an integrated and holistic understanding of the barriers and facilitators to transitional care and the effectiveness of the patient navigator service (How well does it work? What elements of the navigator service were most beneficial to participants? Is it cost saving? How can we scale and disseminate?).

Limitations

There are several limitations to our study design. We are evaluating all cause ER/urgent care visits as the primary outcome. We acknowledge that we are not evaluating avoidable or preventable visits as the primary outcomes, which would intuitively be the outcome of interest. ER/urgent care visits are difficult to categorize in this manner, in particular for the age group being studied. Due to the diversity of eligible patients, participating clinics and sites, there may be a threat to the fidelity of the navigator intervention. We will mitigate this threat by providing study navigators standardized training (lectures, reading material, case based workshops) to develop approaches to address common barriers. An operating manual for the navigator service will be developed from existing work descriptions of navigator staff in pilot programs for diabetes in Alberta, with input from AHS operational managers. Study navigators will receive mentorship and support from pilot program staff. Navigators will meet with team members Samuel and Mackie monthly by conference call to discuss challenges.

Knowledge Translation

This proposal was developed using an integrated knowledge translation approach.[60,61] Our team is comprised of patient representatives, researchers, clinical service providers and senior policy makers who are committed to improving transition and transfer of care within Alberta (see support letters). We developed the intervention and strategy for implementation and evaluation after extensive consultation and engagement with stakeholders in sub-specialty pediatrics and adult chronic disease clinics, emergency medicine, the Well on Your Way Transition Program at ACH, Calgary Zone Primary Care Networks, Calgary Zone Primary Care & Chronic Disease Management Program, Family-Centered Care Team at the SCH, and senior leadership at GRH. We engaged the Child and Youth Advisory Council (a patient council) at the ACH and through a ranking exercise we found that patients valued interventions with personal contact (e.g. patient navigator, peer mentor support) more than those with less personal contact (social media, electronic apps). We will continue to engage patient council groups such as these in Alberta through existing structure of AHS Strategic Clinical Networks. Further, we will consult the Patient and Community Engagement Research Program at the University of Calgary (www.pacerinnovates.ca) to incorporate the patient voice throughout our work, and to guide the interpretation of results and uptake strategies.

Sharing of informational posts on our social media pages will constitute a part of our knowledge translation strategy. This will include information about transition, related resources, and information about publications related to the trial.

EXPERTISE, EXPERIENCE, AND RESOURCES

The Transition Navigator Trial research team is well positioned to do the proposed work. **Dr. Samuel** (Nominated Principal Applicant) is a pediatric nephrologist and clinician scientist (clinical/health

services research, 75% protected time for research) at the University of Calgary. She has a track record of publications and knowledge translation work in transition to adult care; and was the lead author of a transition symposium workshop summary and policy recommendations report to AHS regarding

transition to adult care.[22,23,62] She has substantial expertise in conducting mixed methods multi-centre studies, as she is the Principal Investigator of a CIHR funded national observational study in nephrotic syndrome.[63] She has the necessary research space, computing facilities and access to research methods support teams as needed to coordinate this trial. **Dr. Mackie** (co-Principal Applicant) is a pediatric cardiologist and clinician investigator (40% protected time for research) at the University of Alberta. He is a member of the Women and Children's Health Research Institute (WCHRI), which provides comprehensive research support including data management and biostatistics resources. He has significant expertise in conducting research including intervention trials to improve transition to adult care.[24,42,43] Drs. Samuel and Mackie will jointly supervise a Post-Doctoral Fellow (funded by this grant) in health services research and clinical trials. The Fellow will participate in trial management, data analysis, interpretation and publication of results. **Dr. Dimitropoulos** (co-Principal Applicant) is an Assistant Professor with the Faculty of Social Work at the University of Calgary, and has considerable experience in mixed methods designs, qualitative methodologies and expertise in transition age youth.[64,65] She will perform the qualitative work and analysis with the assistance of one MSc student who will be funded through this grant. **Dr. Nettel-Aguirre** (co-investigator), is a PhD statistician at the University of Calgary; he will lead the analysis for the quantitative data. **Dr. Klarenbach** is a nephrologist and health economist at the University of Alberta and he will guide the cost analysis.

Co-investigators on our team provide depth and breadth of expertise in a variety of clinical disciplines (**Guilcher** -Hematology, **Lang** –Adult Emergency Medicine, **Pacaud**-Diabetes, **Pinzon**-Adolescent Medicine, **Scott**-Nursing/Knowledge Translation, **Zwaigenbaum**-Autism). Collaborators on our team include transition service leaders in Edmonton and Calgary (**Thul, Johnston, Morrison, Andrew**) and a diverse group including patient representatives **(Wilson, Ryan)**, clinical providers and senior policy makers (**Fullerton, Westerlund, Lemarquand-Unich, Taylor, Johnson**) from AHS. Each participating clinic has identified site champions who will assist us to screen for potentially eligible patients using electronic scheduling software used in that location (see collaborators for full list). The team has access to REDCap software to create electronic case report forms at both University of Alberta and University of Calgary, free of charge. The Clinical Analytics team of AHS will provide linked datasets with administrative data relevant to health service utilization outcomes for enrolled patients. Senior AHS leaders (see support letters) in both Edmonton and Calgary have expressed their strong support for this proposal, and will provide work space for the clinical navigators within AHS facilities. The navigators will be hired and managed by AHS managers within pediatric and/or adult care transition services or patient and family oriented care teams in Edmonton and Calgary.

SUMMARY AND SIGNIFICANCE

Transition to adulthood is a complex and challenging time for patients, families, and health care providers. Providing coordinated and seamless transitional care to this vulnerable patient population is a top priority for patients and health service providers in Canada. This study will be the first to test whether a patient navigator implemented across multiple health care settings will reduce expensive ER utilization, and will also help us understand patient, family and provider perceptions of the transition experience, and how a navigator service can support them to improve care. This study will provide urgently needed data to guide pediatric and adult health care providers and policy makers regarding optimal transitional care delivery and will benefit the growing population of young adults living with chronic health conditions.

References

1. Bloom SR, Kuhlthau K, Van Cleave J, Knapp AA, Newacheck P, Perrin JM. Health Care Transition for Youth With Special Health Care Needs. *Journal of Adolescent Health.* 2012;51(3):213-219.
2. Kaufman M, Pinzon, J., Canadian Pediatric Society. Transition to adult care for youth with special health care needs. *Pediatr Child Health.* 2007;12(9):785-788.
3. Betz CL, Redcay G. Lessons learned from providing transition services to adolescents with special health care needs. *Issues in Comprehensive Pediatric Nursing.* 2002;25(2):129-149.
4. Consensus Conference on the Mental Health of Emerging Adults: Making Transitions a Priority in Canada. http://www.mentalhealthcommission.ca/English/consensus-conference-mental-health-emerging-adults.
5. Verma J, Petersen, S., Samis, S.,Akunov, N. and Graham, J. Healthcare Priorities in Canada: A Backgrounder: Canadian Foundation for Healthcare Improvement; 2014.
6. Anthony SJ, Kaufman M, Drabble A, Seifert-Hansen M, Dipchand AI, Martin K. Perceptions of Transitional Care Needs and Experiences in Pediatric Heart Transplant Recipients. *American Journal of Transplantation.* 2009;9(3):614-619.
7. Bindels-de Heus KGCB, van Staa A, van Vliet I, Ewals FVPM, Hilberink SR. Transferring Young People With Profound Intellectual and Multiple Disabilities From Pediatric to Adult Medical Care: Parents' Experiences and Recommendations. *Intellectual and Developmental Disabilities.* 2013/06/01 2013;51(3):176-189.
8. Hendricks DR, Wehman P. Transition From School to Adulthood for Youth With Autism Spectrum Disorders: Review and Recommendations. *Focus on Autism and Other Developmental Disabilities.* March 24, 2009 2009;24(2):77 - 88.
9. Schultz RJ. Parental Experiences Transitioning Their Adolescent With Epilepsy and Cognitive Impairments to Adult Health Care. *Journal of Pediatric Health Care.* 2013;27(5):359-366.
10. McDonagh JE, Viner RM. Lost in transition? Between paediatric and adult services. *BMJ.* 2006-02-23 00:00:00 2006;332(7539):435-437.
11. Bell LE, Bartosh SM, Davis CL, et al. Adolescent Transition to Adult Care in Solid Organ Transplantation: A consensus conference report. *American Journal of Transplantation.* 2008;8(11):2230-2242.
12. McDonagh JE. Growing up and moving on: Transition from pediatric to adult care. *Pediatric Transplantation.* 2005;9(3):364-372.
13. Reiss JG, Gibson RW, Walker LR. Health Care Transition: Youth, Family, and Provider Perspectives. *Pediatrics.* January 1, 2005 2005;115(1):112-120.
14. Por J, Golberg B, Lennox V, Burr P, Barrow J, Dennard L. Transition of care: health care professionals' view. *Journal of Nursing Management.* Sep 2004;12(5):354-361.
15. Okumura MJ, Heisler M, Davis MM, Cabana MD, Demonner S, Kerr EA. Comfort of general internists and general pediatricians in providing care for young adults with chronic illnesses of childhood. *J Gen Intern Med.* Oct 2008;23(10):1621-1627.
16. Suris J-C, Akré C, Rutishauser C. How Adult Specialists Deal with the Principles of a Successful Transition. *Journal of Adolescent Health.* 2009;45(6):551-555.
17. Hovish K, Weaver T, Islam Z, Paul M, Singh SP. Transition experiences of mental health service users, parents, and professionals in the United Kingdom: a qualitative study. *Psychiatric Rehabilitation Journal.* 2012;35(3):251-257.
18. Callahan ST, Cooper WO. Changes in Ambulatory Health Care Use During the Transition to Young Adulthood. *Journal of Adolescent Health.* 2010;46(5):407-413.
19. Van Walleghem N, MacDonald CA, Dean HJ. Evaluation of a Systems Navigator Model for Transition From Pediatric to Adult Care for Young Adults With Type 1 Diabetes. *Diabetes Care.* August 1, 2008 2008;31(8):1529-1530.
20. Prestidge C, Romann A, Djurdjev O, Matsuda-Abedini M. Utility and cost of a renal transplant transition clinic. *Pediatric Nephrology.* Feb 2012;27(2):295-302.

21. Nakhla M, Daneman D, To T, Paradis G, Guttmann A. Transition to Adult Care for Youths With Diabetes Mellitus: Findings From a Universal Health Care System. *Pediatrics.* December 1, 2009 2009;124(6):e1134-e1141.

22. Foster BJ, Dahhou M, Zhang X, Platt RW, Samuel SM, Hanley JA. Association between age and graft failure rates in young kidney transplant recipients. *Transplantation.* Dec 15 2011;92(11):1237-1243.

23. Samuel SM, Nettel-Aguirre A, Hemmelgarn BR, et al. Graft failure and adaptation period to adult healthcare centers in pediatric renal transplant patients. *Transplantation.* Jun 27 2011;91(12):1380-1385.

24. Mackie AS, Ionescu-Ittu R, Therrien J, Pilote L, Abrahamowicz M, Marelli AJ. Children and adults with congenital heart disease lost to follow-up: who and when? *Circulation.* Jul 28 2009;120(4):302-309.

25. Reid GJ, Irvine MJ, McCrindle BW, et al. Prevalence and correlates of successful transfer from pediatric to adult health care among a cohort of young adults with complex congenital heart defects. *Pediatrics.* Mar 2004;113(3 Pt 1):e197-205.

26. Yeung E, Kay J, Roosevelt GE, Brandon M, Yetman AT. Lapse of care as a predictor for morbidity in adults with congenital heart disease. *Int J Cardiol.* Mar 28 2008;125(1):62-65.

27. Cohen E, Gandhi S, Toulany A, et al. Health Care Use During Transfer to Adult Care Among Youth With Chronic Conditions. *Pediatrics.* Mar 2016;137(3):e20152734.

28. Kelly AM, Kratz B, Bielski M, Rinehart PM. Implementing Transitions for Youth With Complex Chronic Conditions Using the Medical Home Model. *Pediatrics.* December 1, 2002 2002;110(Supplement 3):1322-1327.

29. http://www.albertahealthservices.ca/scns/Page11638.aspx.

30. Sawicki GS, Lukens-Bull K, Yin X, et al. Measuring the transition readiness of youth with special healthcare needs: validation of the TRAQ--Transition Readiness Assessment Questionnaire. *J Pediatr Psychol.* Mar 2011;36(2):160-171.

31. Kaufman M, Pinzon J. Transition to Adult Care for Youth with Special Health Care Needs. *Paediatrics & Child Health.* 2007-11 2007;12(9):785-788.

32. Rosen DS, Blum RW, Britto M, Sawyer SM, Siegel DM. Transition to Adult Health Care for Adolescents and Young Adults with Chronic Conditions: Position Paper of the Society for Adolescent Medicine. *Journal of Adolescent Health.* October 2003 2003;33(4):309-311.

33. Annunziato RA, Baisley MC, Arrato N, et al. Strangers headed to a strange land? A pilot study of using a transition coordinator to improve transfer from pediatric to adult services. *Journal of Pediatrics.* Dec 2013;163(6):1628-1633.

34. McDonagh JE, Southwood TR, Shaw KL. The impact of a coordinated transitional care programme on adolescents with juvenile idiopathic arthritis. *Rheumatology (Oxford).* Jan 2007;46(1):161-168.

35. Vanelli M, Caronna S, Adinolfi B, Chiari G, Gugliotta M, Arsenio L. Effectiveness of an uninterrupted procedure to transfer adolescents with Type 1 diabetes from the Paediatric to the Adult Clinic held in the same hospital: eight-year experience with the Parma protocol. *Diabetes Nutr Metab.* Oct 2004;17(5):304-308.

36. Holmes-Walker DJ, Llewellyn AC, Farrell K. A transition care programme which improves diabetes control and reduces hospital admission rates in young adults with Type 1 diabetes aged 15–25 years. *Diabetic Medicine.* 2007;24(7):764-769.

37. Cadario F, Prodam F, Bellone S, et al. Transition process of patients with type 1 diabetes (T1DM) from paediatric to the adult health care service: a hospital-based approach. *Clinical Endocrinology.* 2009;71(3):346-350.

38. Hankins JS, Osarogiagbon R, Adams-Graves P, et al. A transition pilot program for adolescents with sickle cell disease. *J Pediatr Health Care.* Nov-Dec 2012;26(6):e45-49.

39. Hersh A, Pang S, Curran M, Milojevic D, von Scheven E. The challenges of transferring chronic illness patients to adult care: reflections from pediatric and adult rheumatology at a US academic center. *Pediatric Rheumatology.* 2009;7(1):13.

40. Rachas A, Lefeuvre D, Meyer L, et al. Evaluating Continuity During Transfer to Adult Care: A Systematic Review. *Pediatrics.* Jul 2016;138(1).

41. Creswell J, Plano Clark VL., . *Designing and Conducting Mixed Methods Research*. 2nd edition ed: Sage Publications, Inc.; 2011.
42. Mackie AS, Islam S, Magill-Evans J, et al. Healthcare transition for youth with heart disease: a clinical trial. *Heart.* Jul 2014;100(14):1113-1118.
43. Mackie AS, Rempel GR, Kovacs AH, et al. A cluster randomized trial of a transition intervention for adolescents with congenital heart disease: rationale and design of the CHAPTER 2 study. *BMC Cardiovasc Disord.* 2016;16:127.
44. Rempel GR, Ballantyne RT, Magill-Evans J, Nicholas DB, Mackie AS. Texting teens in transition: the use of text messages in clinical intervention research. *JMIR Mhealth Uhealth.* 2014;2(4):e45.
45. Sevick MA, Trauth JM, Ling BS, et al. Patients with Complex Chronic Diseases: perspectives on supporting self-management. *J Gen Intern Med.* Dec 2007;22 Suppl 3:438-444.
46. Major J, Stewart, D., Amaria, K., Nguyen, T., Doig, J., Adams, S., Giroux, C., Freeman, M., Samis, S., Kaufman, M., Gorter, J.W., Gaffney J.B., Wilson, L. Care in the Long Term for Youth and Young Adults with Complex Care Needs. Ottawa: Canadian Foundation for Healthcare Improvement; 2014.
47. Betz CL, Redcay G. Dimensions of the Transition Service Coordinator Role. *Journal for Specialists in Pediatric Nursing.* 2005;10(2):49-59.
48. Billings J, Zeitel L, Lukomnik J, Carey TS, Blank AE, Newman L. Impact of socioeconomic status on hospital use in New York City. *Health Aff (Millwood).* Spring 1993;12(1):162-173.
49. Chan AW, Tetzlaff JM, Altman DG, Dickersin K, Moher D. SPIRIT 2013: new guidance for content of clinical trial protocols. *Lancet.* Jan 12 2013;381(9861):91-92.
50. Chan AW, Tetzlaff JM, Altman DG, et al. SPIRIT 2013 statement: defining standard protocol items for clinical trials. *Ann Intern Med.* Feb 5 2013;158(3):200-207.
51. Chan AW, Tetzlaff JM, Gotzsche PC, et al. SPIRIT 2013 explanation and elaboration: guidance for protocols of clinical trials. *BMJ.* 2013;346:e7586.
52. Baxter PaJ, S. Qualitative Case Study Methodology: Study Design and Implementation for Novice Researchers. *The Qualitative Report.* December 2008 2008;13(4):544-559.
53. Yin RK, ed *Case Study Research: Design and Methods*: Sage; 2003; No. Volume 5 of Applied Social Research Methods.
54. Braun VaC, V. Using thematic analysis in psychology. *Qualitative Research in Psychology.* 2006;3(2):77-101.
55. Krueger RAaC, M.A. *Focus groups: A Practical Guide for Applied Research*. 5 ed: Sage Publications; 2014.
56. Creswell J, ed *Qualitative Evaluation and Research Methods.* 3rd Edition ed. Newbury Park, CA: Sage; 2002.
57. Elliott R, Fischer CT, Rennie DL. Evolving guidelines for publication of qualitative research studies in psychology and related fields. *Br J Clin Psychol.* Sep 1999;38 (Pt 3):215-229.
58. Gold MR, Siegel, J., Russell, L.B., et al. . *Cost Effectiveness in Health and Medicine*. New York: Oxford University Press; 1996.
59. Duan N. Smearing estimate: a nonparametric transformation method. *J Am Stats Assoc.* 1983;78:605-610.
60. Straus SE, Tetroe J, Graham I. Defining knowledge translation. *CMAJ.* Aug 4 2009;181(3-4):165-168.
61. Graham ID, Logan J, Harrison MB, et al. Lost in knowledge translation: time for a map? *J Contin Educ Health Prof.* Winter 2006;26(1):13-24.
62. Samuel SM, Nettel-Aguirre A, Soo A, Hemmelgarn B, Tonelli M, Foster B. Avoidable hospitalizations in youth with kidney failure after transfer to or with only adult care. *Pediatrics.* Apr 2014;133(4):e993-1000.
63. Samuel S, Scott S, Morgan C, et al. The Canadian Childhood Nephrotic Syndrome (CHILDNEPH) Project: overview of design and methods. *Can J Kidney Health Dis.* 2014;1:17.
64. Dimitropoulos G, Tran AF, Agarwal P, Sheffield B, Woodside B. Navigating the transition from pediatric to adult eating disorder programs: perspectives of service providers. *International Journal of Eating Disorders.* Sep 2012;45(6):759-767.

65. Dimitropoulos G, Tran AF, Agarwal P, Sheffield B, Woodside B. Challenges in making the transition between pediatric and adult eating disorder programs: a qualitative study from the perspective of service providers. *Brunner-Mazel Eating Disorders Monograph Series.* 2013;21(1):1-15.
