# Supplementary material for: Effectiveness of Patient Navigation During Transition to Adult Care: A Randomized Clinical Trial
Source: JAMA Pediatr. 2025 Feb 10;179(4):375–82. doi: 10.1001/jamapediatrics.2024.6192 (PMC11811865; doi:10.1001/jamapediatrics.2024.6192)
Supplement: Supplement 2. — Data Sharing Statement. [file jamapediatr-e246192-s002.pdf]

Data Sharing Statement

Samuel. Effectiveness of Patient Navigation During Transition to Adult Care. *JAMA Pediatr*. Published February 10, 2025. doi:10.1001/jamapediatrics.2024.6192

Data
Additional Information: Clinicaltrials.gov Trial registration: NCT03342495
Data available: No

Additional Information
Explanation for why data not available: We need additional approvals to make this happen.
